# Semiautomatic volumetry of the temporal lobes of the brain and correlation with electroencephalography results in dogs with assumed idiopathic epilepsy

**DOI:** 10.1111/jvim.17237

**Published:** 2024-11-04

**Authors:** Paulina Kinga Drobot, Aleksandra Banasik, Karolina Owsińska‐Schmidt, Marcin Wrzosek, Przemysław Podgórski

**Affiliations:** ^1^ Department of Internal Medicine and Clinic for Horses, Dogs and Cats The Faculty of Veterinary Medicine, Wrocław University of Environmental and Life Sciences Wroclaw Poland; ^2^ Division of General and Interventional Radiology and Neuroradiology, Department of Radiology Faculty of Medicine, Wroclaw Medical University Wroclaw Poland

**Keywords:** asymmetric ratio, dog, epileptiform discharges, magnetic resonance imaging, radiology and diagnostic imaging, temporal lobe epilepsy

## Abstract

**Background:**

Lesions causing refractory epilepsy, often associated with temporal lobe epilepsy (TLE), can be undetectable on standard magnetic resonance imaging (MRI) in dogs. Automated brain volumetry, widely used in human medicine, can now be applied in veterinary medicine because of the availability of brain atlases.

**Objectives:**

This study aimed to develop an automatic volumetry method, translate the outcomes into the assessment of temporal lobe volumes in dogs with idiopathic epilepsy, and correlate the results with the electroencephalography (EEG) data of epileptiform discharges (EDs).

**Animals:**

Thirty‐one dogs of various breeds with dominant temporal lobe discharge.

**Methods:**

Retrospective, observational study. The MRI and EEG examination results of dogs referred for neurological diagnosis data between 2016 and 2021 were retrospectively analyzed. An automated volumetry method was developed, which allowed the evaluation of temporal lobe volumes of the dogs. The asymmetric ratio (AR) was then estimated, and the results were correlated with the EEG EDs.

**Results:**

12/31 (38%; 95% CI: 21.8%‐57.8%) dogs had an asymmetric ratio >6%. Among them, reduction in temporal lobe volumes correlated with the side of the EEG EDs in 7 cases. There was no statistical correlation between temporal lobe volume changes and ED location.

**Conclusions and Clinical Importance:**

Preliminary volumetric analysis of the temporal lobes indicates the presence of volume differences between the lobes in some dogs with idiopathic epilepsy. Diagnosis of TLE in dogs based on MRI volumetry in correlation with EEG examination, especially for dogs with drug‐resistant epilepsy, can influence the development of new therapeutic options, such as surgery.

**Abbreviations**
EDs: epileptiform discharges
EEG: electroencephalography
IVETF: International Veterinary Epilepsy Task Force
MRI: magnetic resonance imaging
TL: temporal lobe
TLE: temporal lobe epilepsy

## 1 INTRODUCTION

Epilepsy is one of the most common chronic neurological diseases in dogs with a prevalence of approximately 0.75%.^1^, ^2^ According to the International Veterinary Epilepsy Task Force (IVETF) consensus, the umbrella term “idiopathic epilepsy” comprises 3 main categories of seizure etiology: (1) genetic epilepsy, (2) suspected genetic epilepsy, and (3) epilepsy of unknown cause.^3^ Seizures can take different courses and are not always characterized by generalized tonic‐clonic seizures.^4^ Thus, electroencephalography (EEG), an important diagnostic tool in human medicine, could help diagnose epilepsy in dogs, allowing us to confirm abnormal brain activity by visible epileptiform discharges (EDs) and their locations.

Temporal lobe epilepsy (TLE), one of the most frequently diagnosed forms of epilepsy in humans,^5^, ^6^ is described in dogs, mostly based on hippocampal sclerosis and atrophy.^7^, ^8^ Other structures such as the temporal lobe and amygdala have not been evaluated. Notably, a standard magnetic resonance imaging (MRI) of the brain and EEG could not be sufficient to distinguish between TLE and other forms of epilepsy classified as pure idiopathic epilepsy. A more accurate assessment, especially of the brain lobes or small structures, is possible using a volumetric analysis.

Automated brain volumetry is a developed tool in human medicine,^9^, ^10^ but in dogs, owing to the diversity of species, its automation and analysis of some small structures in the brain have not been possible owing to lack of proper software and population atlases. Until now, most of the analyses have been performed manually, which is associated with low sensitivity. Owing to rapid advances in veterinary medicine, brain atlases are now available and can be used in the volumetry of individual brain structures such as the gray matter, white matter, and brain lobes.^11^, ^12^ Accordingly, an automated method of brain volumetry can be developed to generate more accurate results. The first goal of the present study was to develop an automated volumetric method based on available canine brain atlases.^11^ The second goal was the assessment of the dependency between temporal lobe EDs on EEG with temporal lobe volumetry data.

## 2 MATERIALS AND METHODS

This study aimed to develop an automatic volumetry method, translate the outcomes into the assessment of temporal lobe volume in dogs with idiopathic epilepsy, and correlate the results with the EEG recordings of EDs.

### 2.1 Case selection

This retrospective study was conducted using the MRI and EEG examination results of dogs referred for neurological consultation and EEG at the Clinic for Horses, Dogs, and Cats at the Department of Internal Medicine at the University of Environmental and Life Sciences in Wrocław, Poland, between 2016 and 2021. The MRI studies were performed at the Center for Experimental Diagnostics and Biomedical Innovations at the same institution. All MRI and EEG recordings were obtained with the client consent.

### 2.2 Inclusion criteria

Dogs with epilepsy diagnosed using the criteria proposed by the IVETF consensus statement (Tier I‐III)^13^ with dominant epileptic discharges on the temporal leads were included in the study. Dogs with severe asymmetry and dilatation of the ventricular system, in which Bh/Hv and Bw/Vw indicated mild to severe ventriculomegaly, were excluded from the study.^14^

### 2.3 EEG recording

**FIGURE 1:**
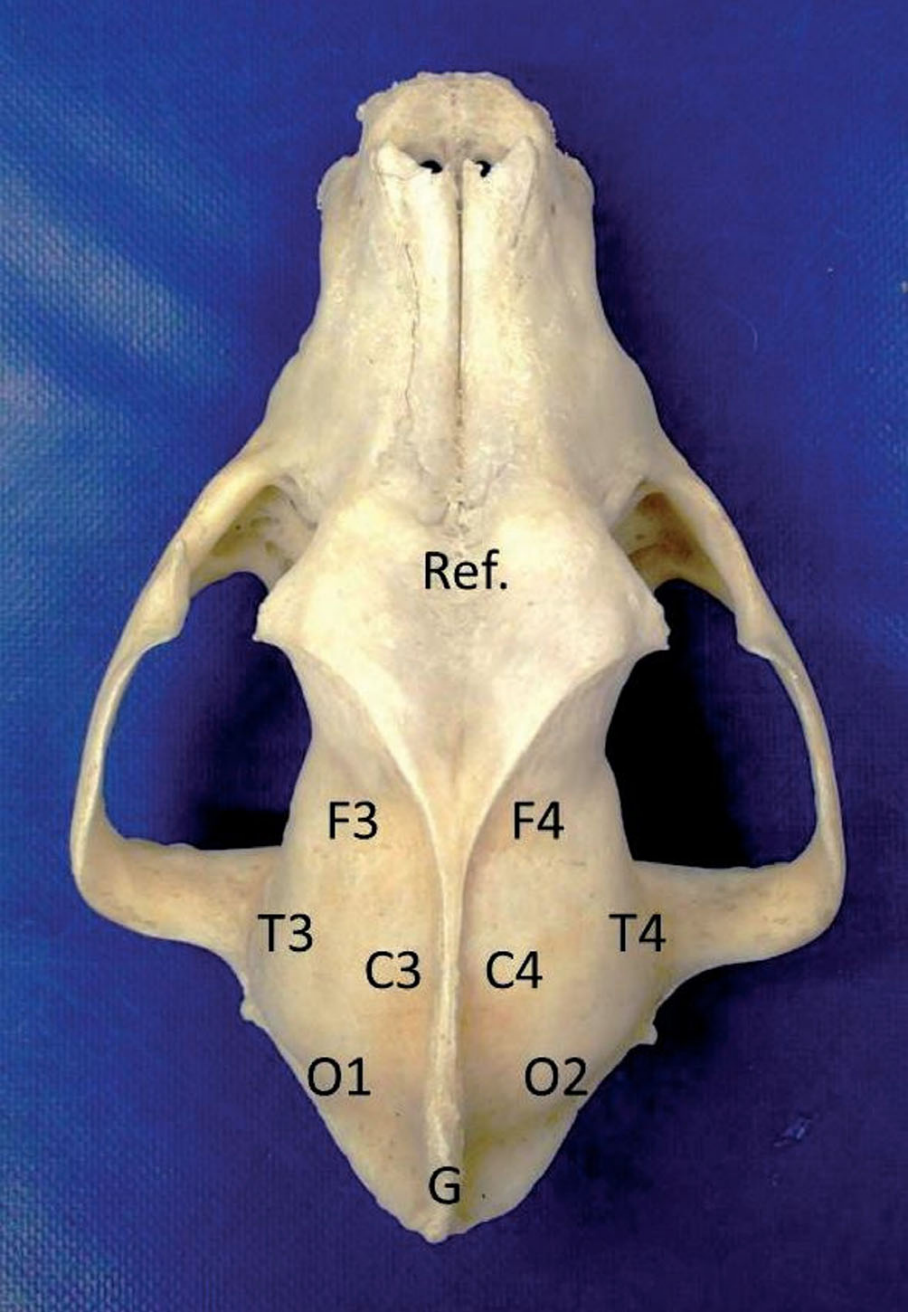
Diagram of electrode placement for EEG examination on a canine skull model.

The EEG recordings were performed under sedation using medetomidine at a dose of 20 mcg/kg injected intramuscularly (IM). The dogs included in the study were placed in the sternal position, and the entire EEG recording was video‐recorded to eliminate movement artifacts. A 30‐minute protocol developed by one of the authors (MW) was used,^15^ and a Nihon Kohden EEG (Nihon Kohden Corporation, Tokyo, Japan) unit with the following settings was applied with a 60 Hz notch filter inserted: sensitivity, 70 μV/cm; bandpass filter, 0.5 to 30.0 Hz; and time constant, 0.3 second. The EEG recordings were performed using subcutaneous lead electrodes, and each recording was made using a 10‐channel reference montage (F3, F4, C3, C4, T3, T4, O1, O2‐Ref., the reference electrode was placed on the frontal bone, the substrate electrode in the neck, and a standard bipolar montage [F3‐C3, C3‐T3, T3‐O1, F4‐C4, C4‐T4, T4‐O2]; Figure 1). The ECG‐Ref. electrode was placed subcutaneously at level 5 of the intercostal space on the left side, near the chondrocostal junction. Light stimulation was performed 10 minutes later using a stroboscope lamp. The initial stimulation frequency was 0.5 Hz, which was gradually increased to 60 Hz and then steadily decreased to baseline over 5 minutes, according to the protocols described in the veterinary literature.^15^, ^16^ Visual analyses of the EEG results were performed by 2 authors (PD, MW) simultaneously using monopolar and bipolar montage in correlation with video recordings.

### 2.4 MRI study

Immediately after the EEG test, the dogs were transported for the MRI. Anesthesia was induced using propofol (3‐5 mg/kg; Propofol Lipuro, 10 mg/mL, B Braun, Melsungen AG) and was maintained after intubation using inhaled isoflurane in oxygen (1.5‐2%vol). Structural brain MRI was performed using a 1.5‐T scanner (Ingenia, Philips Healthcare, Eindhoven, the Netherlands) with an 8‐channel head coil. The following sequences were used to scan the brains of the dogs: 3D T1‐W, T2‐W sagittal, transverse, FLAIR transverse, T2*transverse, and 3D T1W with a contrast agent.

A T1‐3D volumetric sequence was included in the scanning protocol (field of view, 200 × 200 mm; matrix, 268 × 266; voxel size, 0.75 × 0.75 × 0.75 mm; flip angle, 30°; repetition time [TR] = 25 ms; echo time [TE] = 4.9 ms).

### 2.5 MR volumetry

The first stage of the analysis consisted of utilizing manual semiautomated techniques for brain segmentation. The segmentation was performed using MRIcron software. Several manual 3D Volume of Interest (VOI) selections were made based on background intensity to define regions of interest, such as the brain, cerebellum, and spinal cord. The parameters, including the radius of the volume of interest (VOI), the number of erosion/dilation cycles, and the intensity differences between the origin and edge, were carefully fine‐tuned to accurately isolate the desired tissues and exclude any unnecessary structures.

**FIGURE 2:**
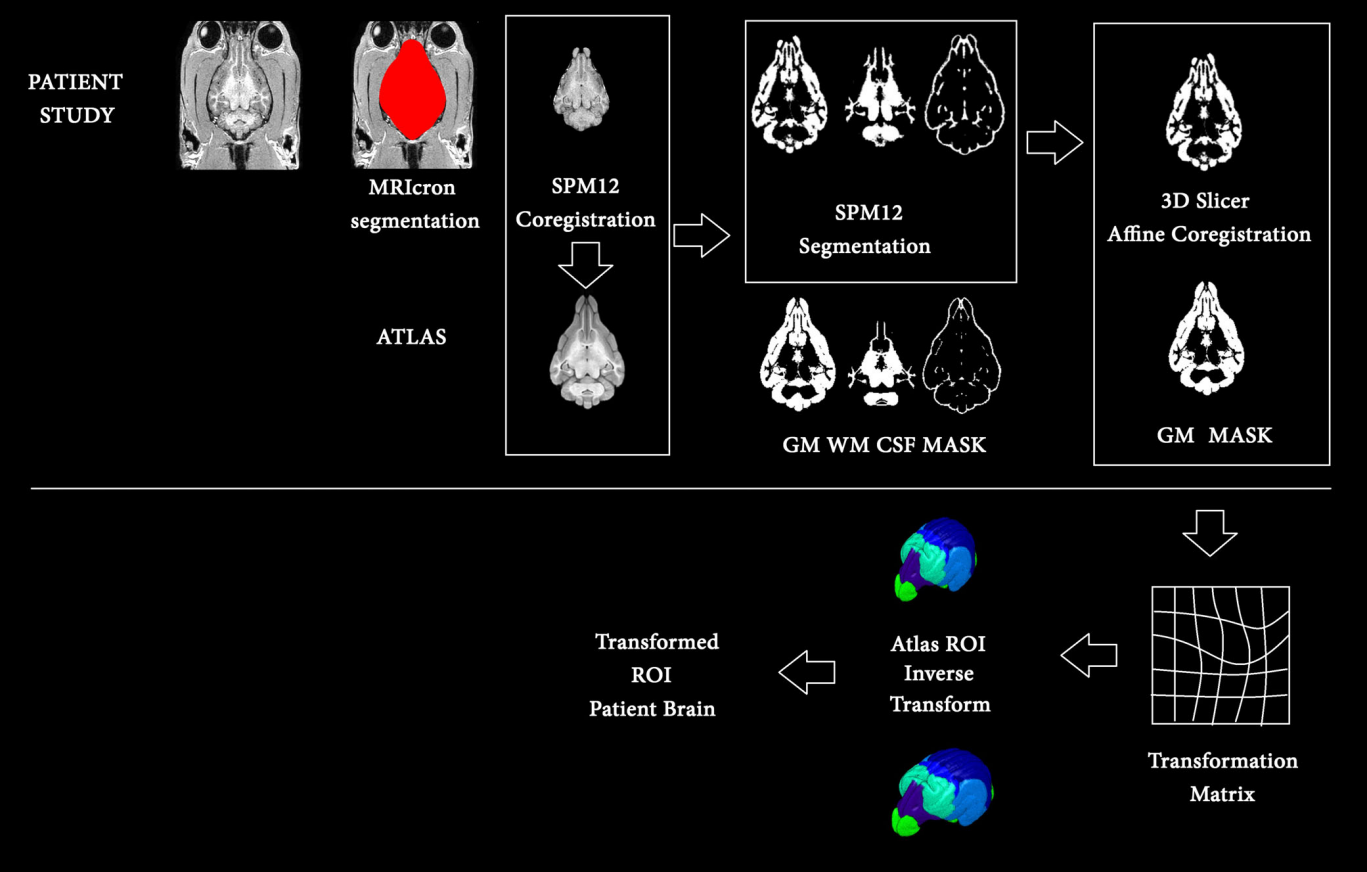
Visualization of brain segmentation before correlation with the dog atlas and a comparison with the atlas results overlaid.

Afterward, the brain that was extracted underwent coregistration with a stereotactic Cortical Atlas of the Domestic Canine Brain using Statistical Parametric Mapping 12 (SPM12). The process entailed registering the data to the atlas space, subsequently dividing it into gray matter, white matter, and cerebrospinal fluid through segmentation. The affine registration functionality of 3D Slicer was employed to accurately align each dataset with the atlas, guaranteeing spatial precision (Figure 2).

**FIGURE 3:**
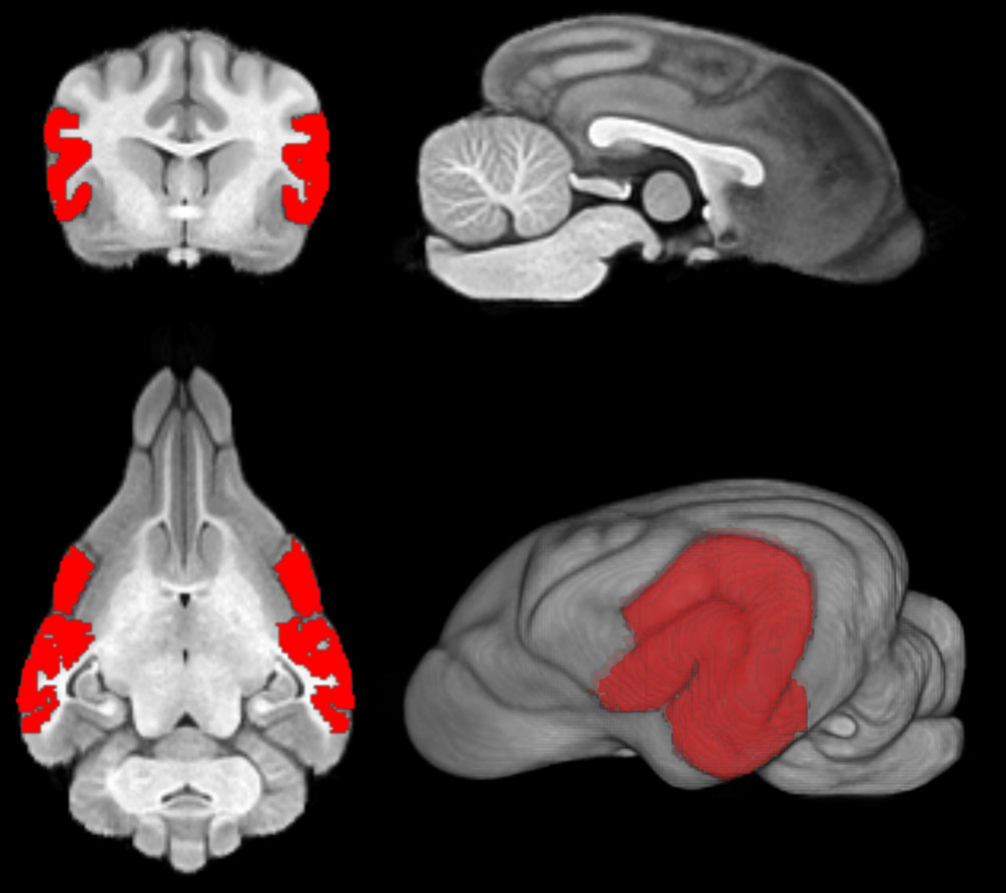
Visualization of the matched temporal lobes on MRI in T1‐weighted sequence in 3‐dimensional planes and 3D reconstruction.

The acquired Jacobian matrix was subsequently employed in an inverse fitting procedure to precisely align each region of interest with the corresponding canine brain structure. The volume of the fitted temporal lobe VOI closely matched the volume of the individual subject's temporal lobe, confirming the accuracy of the segmentation and registration processes (Figure 3).

The asymmetric ratio was estimated from the difference in the volume of the larger and smaller temporal lobe, divided by the volume of the larger temporal lobe and multiplied × 100%. According to the available literature, a cut‐off value for asymmetry was set >6%. This calculation was aligned with the 6% asymmetry observed in hippocampal volume studies, providing a comparative benchmark for evaluating temporal lobe asymmetry.^7^, ^17^, ^18^

### 2.6 Data analysis

The first analysis involved assessing the correlation between temporal lobe volume changes and the location of discharges on the EEG. The animals were divided into 3 groups according to the site of EDs on EEG as follows: T4, Group A; T3, Group B; and T3 and T4, Group C. The differences in asymmetric ratio (AR) values, right temporal lobe volume (vTR), and left temporal lobe volume (vTL) between the groups were analyzed.

Both the presence of EDs and subjective ventricular assessments in classical MRI sequences were the variables that were nonnormally distributed.

Therefore, the acquired data on the presence of discharges and the morphology of the ventricular system were used to make interclass divisions within the study group and perform analyses to assess the statistical significance of the acquired volumetric data. To complete the above analysis, the animals were divided according to the radiological changes of the ventricular system into the following 3 groups: L > R, Group 1; R > L Group 2; and symmetric ventricles, Group 3. Differences in AR, vTR, and vTL values between the groups were analyzed.

Statistical analyses were performed using the commercial software system Statistica 13.3 (TIBCO Software, Inc, Palo Alto, CA, 2017).

Testing for the normal distribution of the data was performed using the Shapiro‐Wilk normality test. Assumptions regarding the normality of the data distribution were maintained in the analyzed cases.

For values that did not meet the assumptions of a normal distribution (nonparametric values), the Kruskal‐Wallis rank ANOVA test was used to assess the differences between the average values of AR, vTR, and vTL between the groups. To highlight the differences between the groups, multiple comparisons of the mean ranks for all groups were performed using the multiple comparison post hoc test. Multiple comparison Wilcoxon test was a post hoc test that was used for analyses showing statistically significant differences.

## 3 RESULTS

### 3.1 Study group characteristics

Thirty‐one dogs with temporal lobe discharge were enrolled in this study. The group included 22 males and 9 females, consisting of 15 pure breeds (23 dogs; 4 border collies, 3 Labrador retrievers, 2 beagles, 2 coton de Tulear, 2 golden retrievers, 1 German pointer, 1 giant schnauzer, 1 Samoyed, greater Swiss mountain dog, 1 Maltipoo, 1 Polish hunting dog, 1 Polish lowland sheepdog, 1 Siberian husky, 1 small Munsterlander, and 1 Yorkshire Biewer), and 8 mixed‐breed dogs. All dogs underwent routine neurological examinations and blood tests. The types of ictal signs observed in the dogs were as follows: generalized seizures (n = 25), focal seizures (n = 3), and focal seizures turning into generalized seizures (n = 3). At the time of EEG examination, 7 dogs were not prescribed antiepileptic drugs. The prescribed antiepileptic drugs for the other dogs were: phenobarbital (n = 10); imepitoin (n = 5); phenobarbital and gabapentin (n = 2); phenobarbital and levetiracetam (n = 2); phenobarbital and potassium bromide (n = 1); imepitoin and potassium bromide (n = 1); phenobarbital, potassium bromide, and clonazepam (n = 1); phenobarbital, potassium bromide, and gabapentin (n = 1); and phenobarbital, potassium bromide, gabapentin, and levetiracetam (n = 1).

### 3.2 EEG results

**FIGURE 4:**
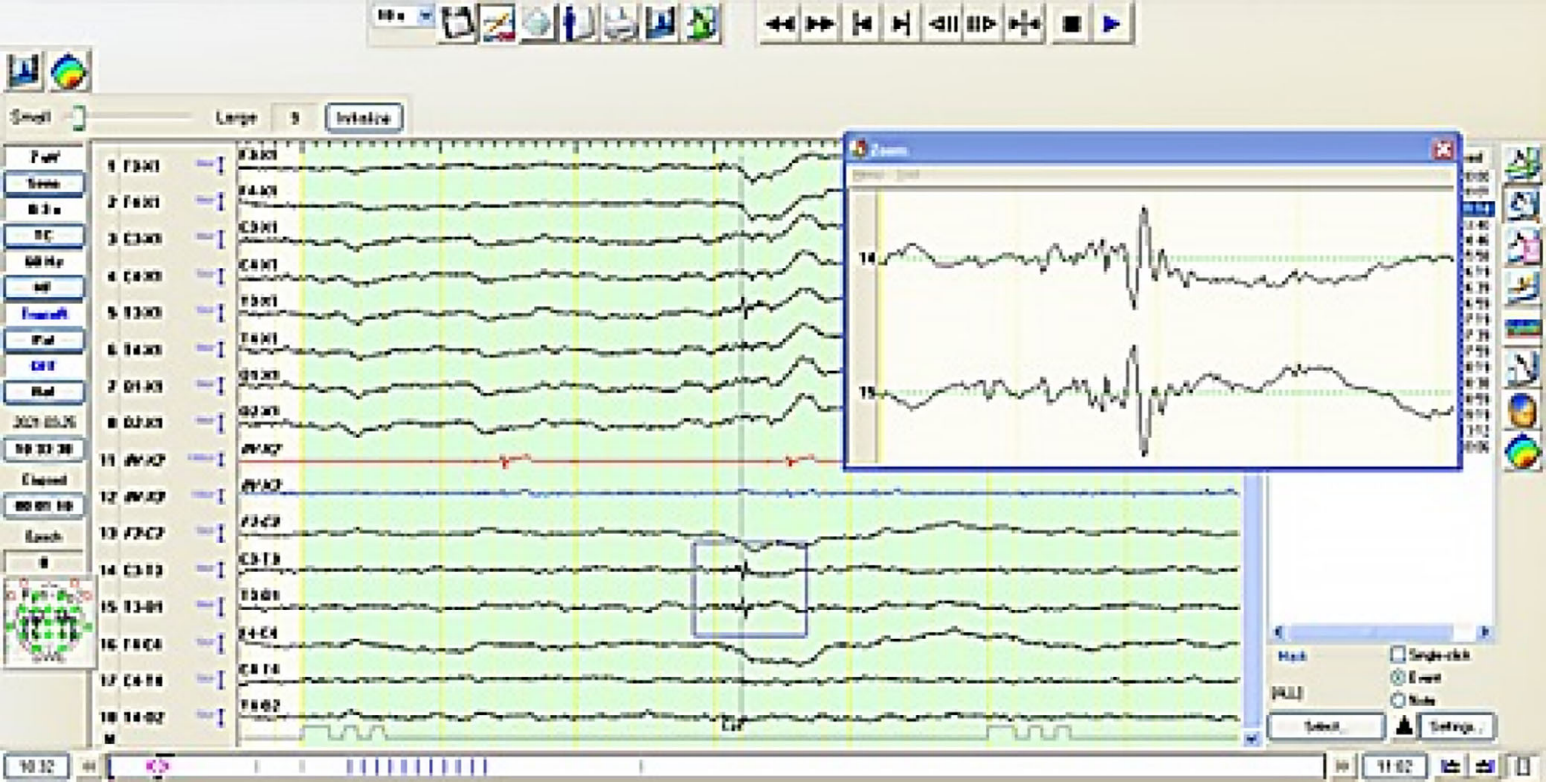
Electroencephalographic recordings from a German pointer showing spike activity and polyspike activity from the T3 lead.

In total, 14/31 dogs (45%; 95 CI: 27.7%‐62.7%) had EDs in T4 leads (Group A), 12 (38%; 95% CI: 21.8%‐57.8%) had EDs in T3 leads (Group B), and 5 (16%; 95% CI: 3.8%‐29%) had EDs in both T3 and T4 leads (Group C; Figure 4).

### 3.3 MRI findings

Of the 31 dogs, 26 (84%; 95% CI: 71%‐96.8%) showed ventricular asymmetry on MRI. Of these, 19/31 (61%; 95% CI: 44.1%‐78.4%) dogs showed a larger left ventricle, and 7/31 (23%; 95% CI: 7.9%‐37.3%) dogs showed a larger right ventricle.

### 3.4 Volumetric measurements and asymmetric ratio findings

Left temporal lobe volumes ranged from 3932.29 to 5026.3 mm^3^ (median: 4669.93 mm^3^, mean: 4572.2 mm^3^), and right temporal lobe volumes ranged from 3612.22 to 5012.68 mm^3^ (median: 4723.21 mm^3^, mean: 4601.79 mm^3^).

**FIGURE 5:**
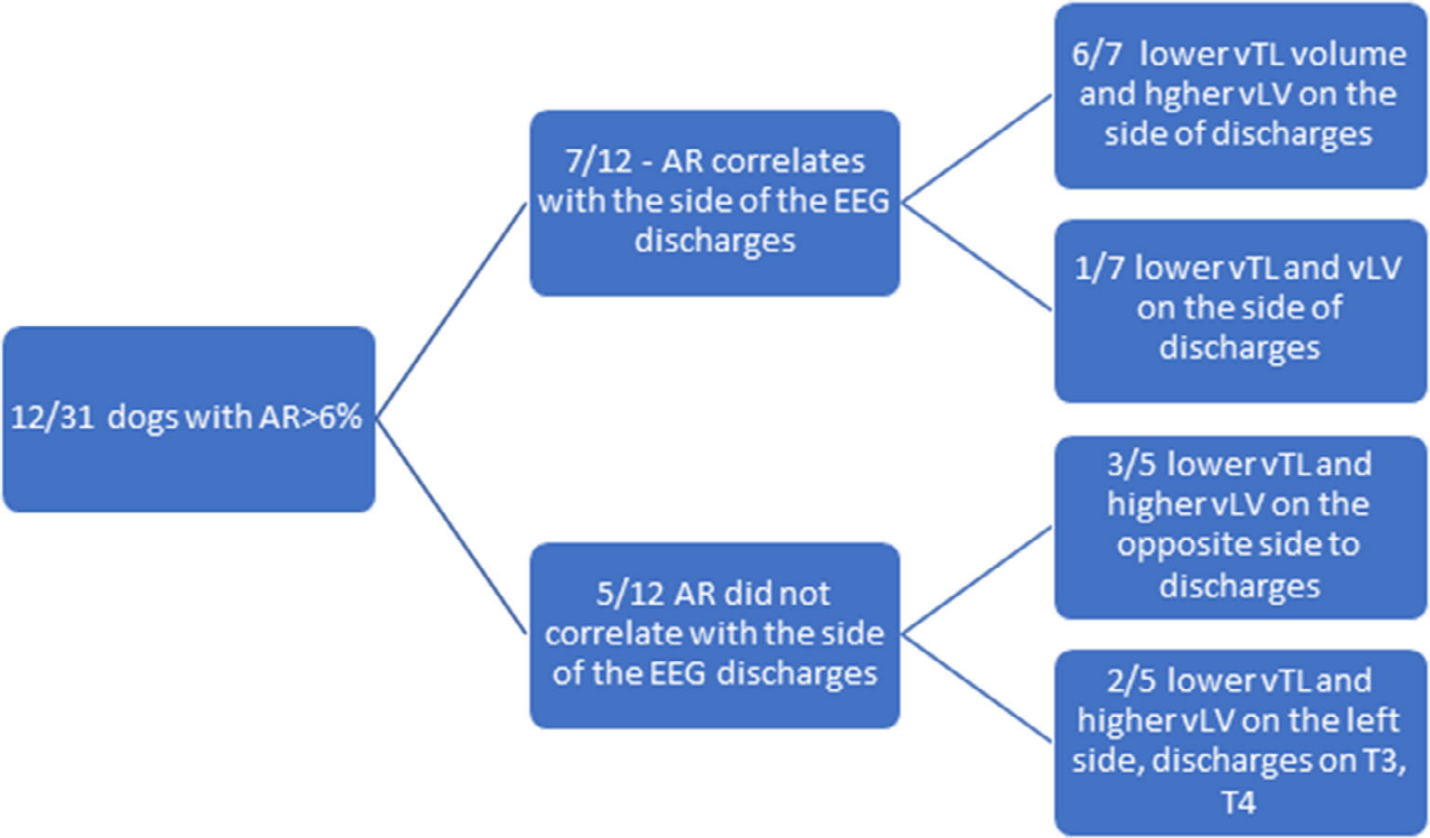
Distribution of dogs with asymmetric ratio (AR) above 6%. vTL, temporal lobe volume; vLV, lateral ventricle volume.

Additionally, 12/31 (38.7%; 95% CI: 21.8%‐57.8%) dogs had an AR above 6%, of which 6 dogs (50%; 95% CI: 21.7%‐78.3%) showed a correlation between the ED side and a reduction in temporal lobe volume and widening of the lateral ventricle. In the remaining 6 of 12 dogs, 3 (50%) had decreased temporal lobe volumes and enlarged lateral ventricles on the side opposite to the EDs; 1 had a decreased temporal lobe volume and decreased lateral ventricle volume on the side of EDs; and 2 had decreased temporal lobe volumes, enlarged lateral ventricles on the left side, and bilateral EDs (T3 and T4; Figure 5).

**FIGURE 6:**
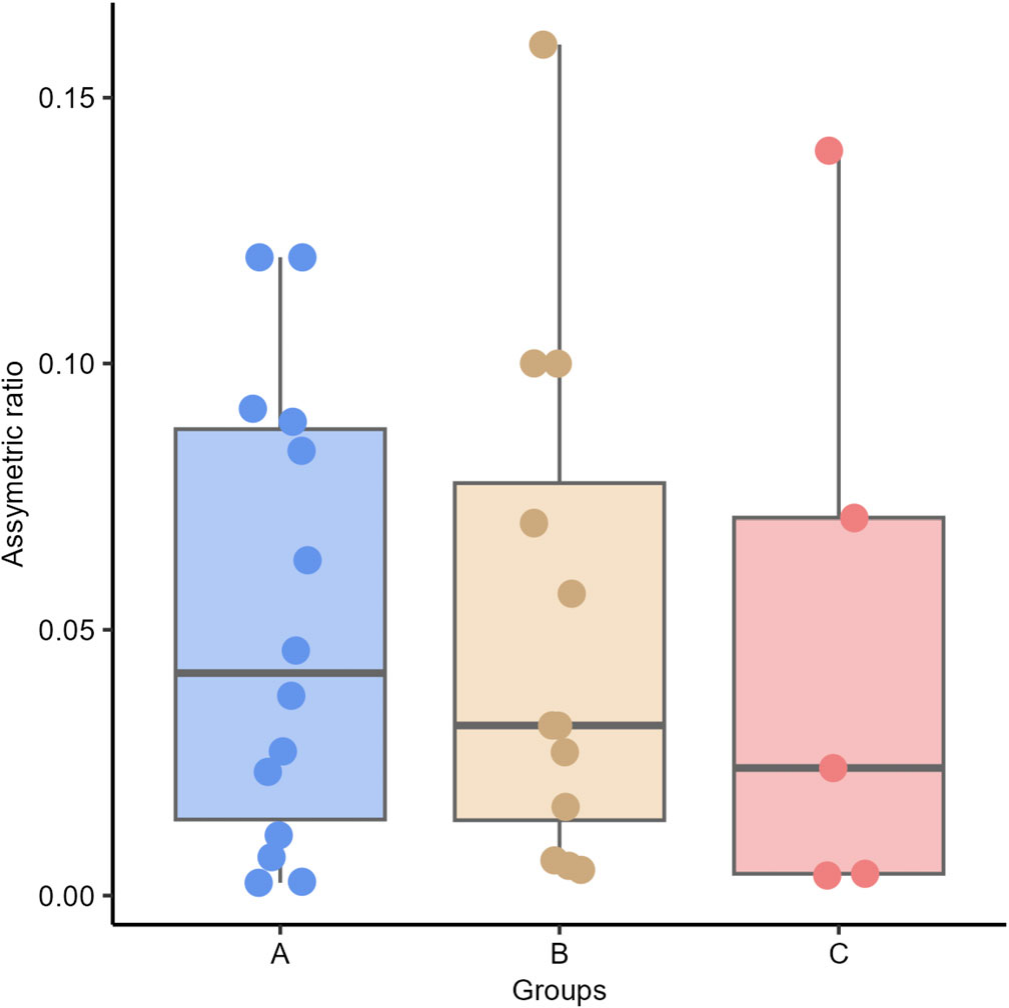
Box‐and‐whisker plots and individual data points show distribution of the asymmetric ratio (AR) between Groups A (group of dogs with a discharge in T3), B (T4), and C (T3 and T4).

**FIGURE 7:**
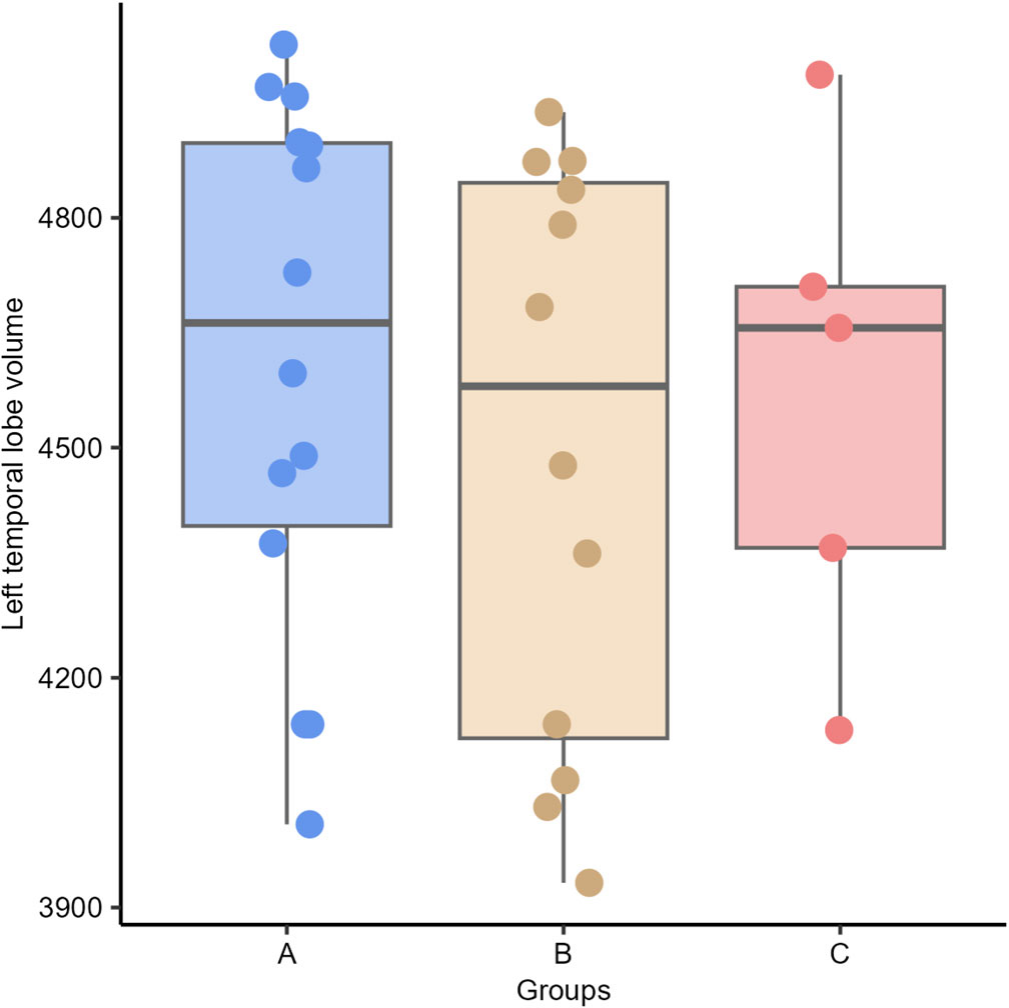
Box‐and‐whisker plots and individual data points show distribution of the left temporal lobe volumes (vTL) between Groups A (group of dogs with a discharge in T3), B (T4), and C (T3 and T4).

**FIGURE 8:**
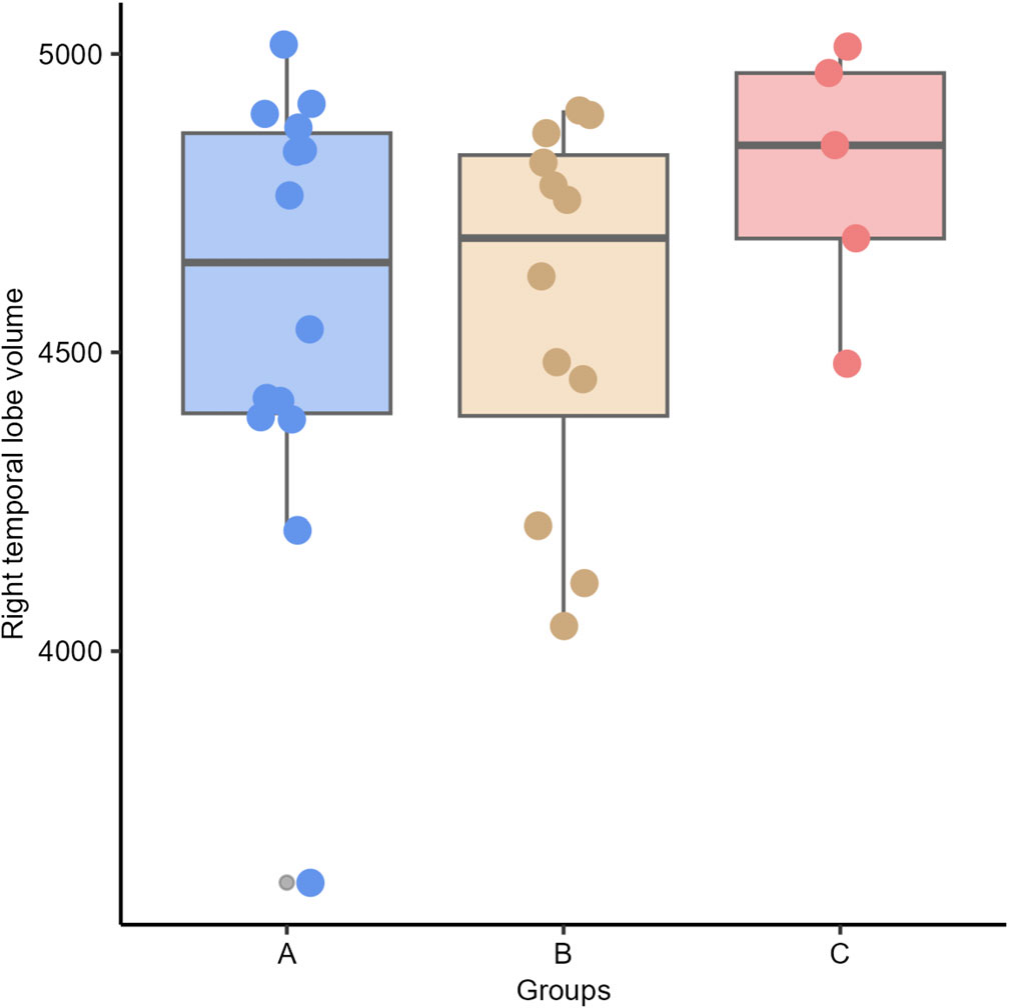
Box‐and‐whisker plots and individual data points show distribution of the right temporal lobe volumes (vTR) between Groups A (group of dogs with a discharge in T3), B (T4), and C (T3 and T4).

No differences were found between groups in both, left and right temporal lobe volumes (*P* = .610 and *P* = .340, respectively) and AR (*P* = .89) between groups: A (group of dogs with a discharge in T3), B (T4), and C (T3 and T4; Figures 6, 7, 8).

**FIGURE 9:**
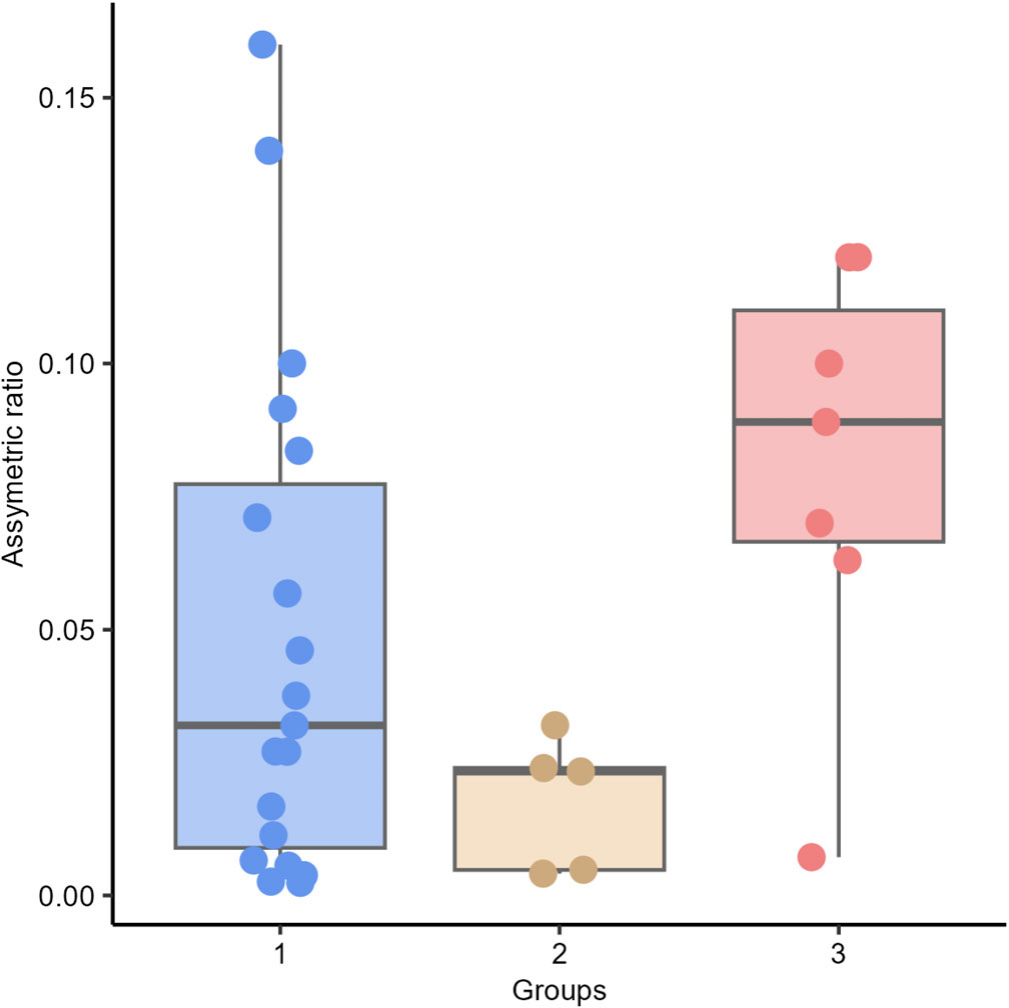
Box‐and‐whisker plots and individual data points show distribution of the asymmetric ratio (AR) between Groups 1 (left lateral ventricle larger than right), 2 (right lateral ventricle larger than the left), and 3 (symmetric lateral ventricle).

**FIGURE 10:**
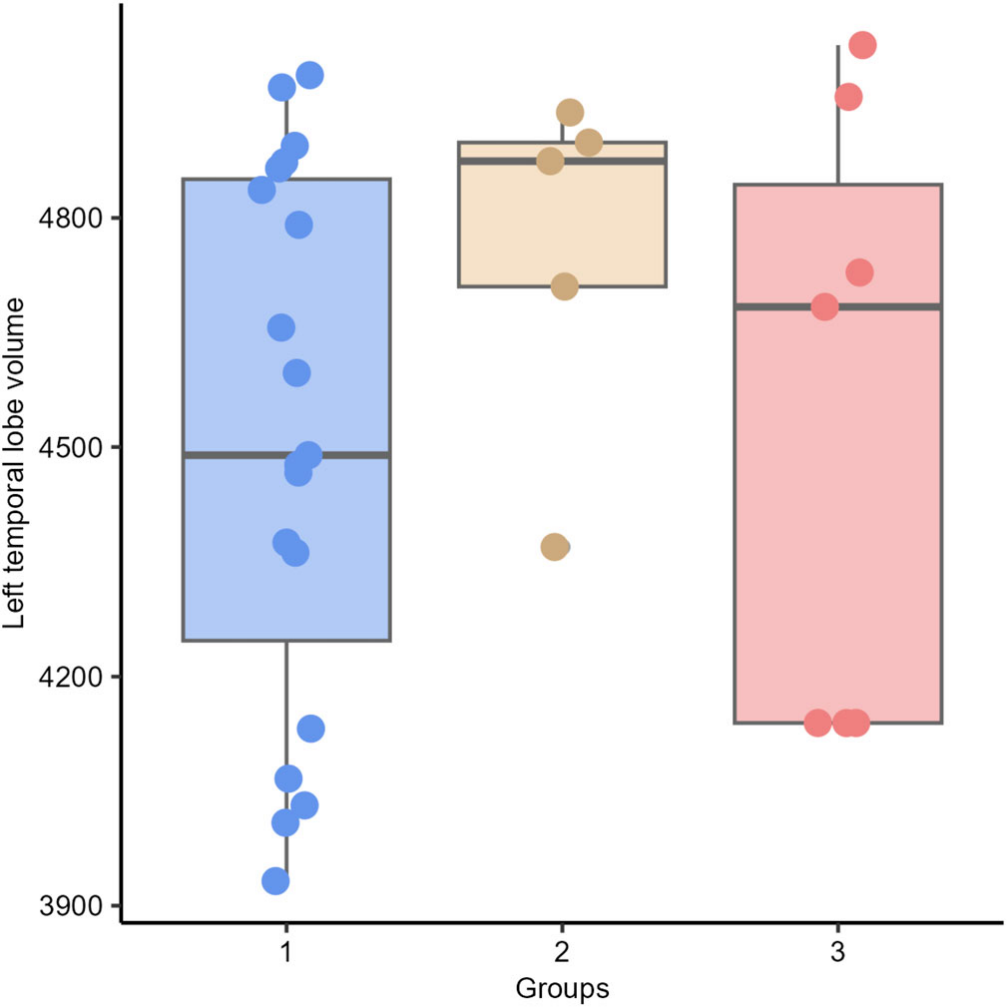
Box‐and‐whisker plots and individual data points show distribution of the left temporal lobe volume (vTL) between Groups 1 (left lateral ventricle larger than right), 2 (right lateral ventricle larger than the left), and 3 (symmetric lateral ventricle).

**FIGURE 11:**
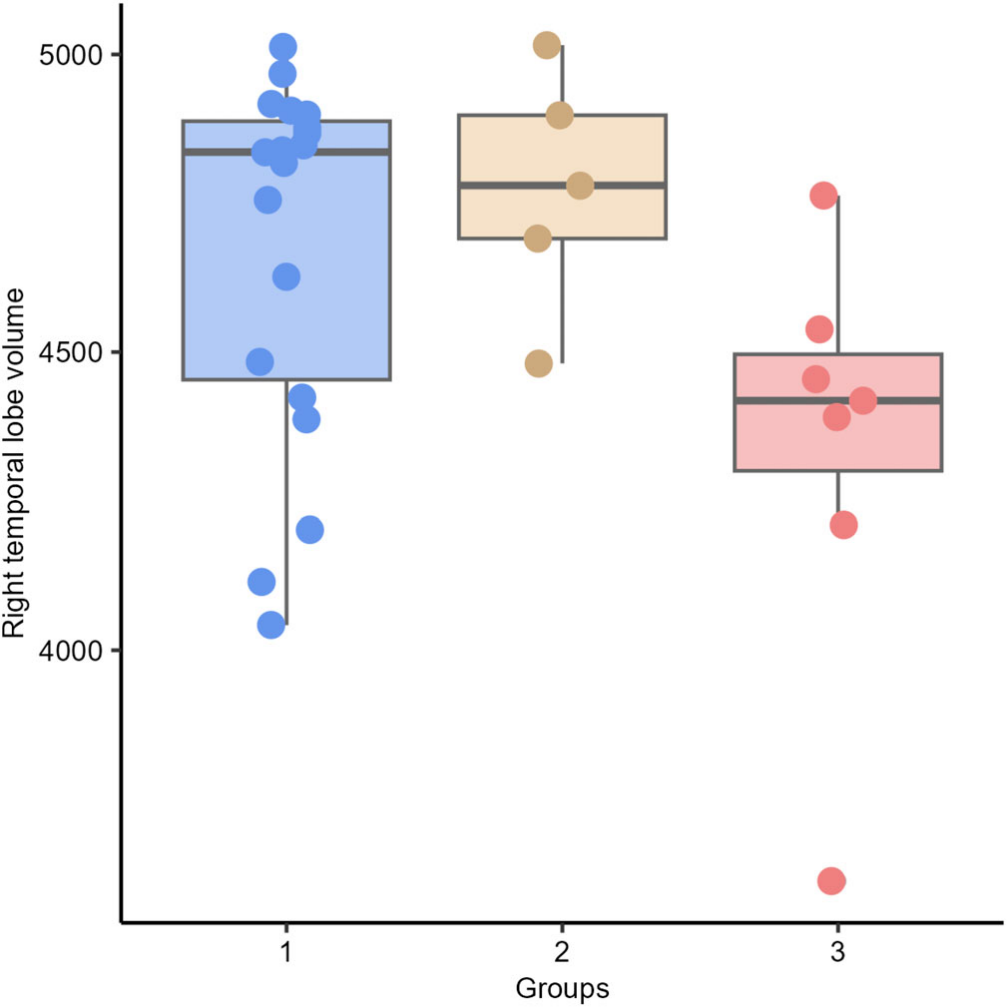
Box‐and‐whisker plots and individual data points show distribution of the right temporal lobe volume (vTR) between Groups 1 (left lateral ventricle larger than right), 2 (right lateral ventricle larger than the left), and 3 (symmetric lateral ventricle).

We observed significant differences in AR between Groups 2 (right lateral ventricle larger than the left) and 3 (symmetric lateral ventricle [*P* = .047]). No significant differences were observed among the other groups (between Groups 1 [left lateral ventricle larger than right] and 2 [*P* = .597]; between Groups 1 and 3 [*P* = .246]; Figure 9). There were no statistically significant differences in left (*P* = .354) and right temporal lobe volumes (*P* = .080) between Groups 1, 2, and 3 (Figures 10 and 11).

Descriptive statistics with effect sizes for analyses performed are provided in Supporting Information.

## 4 DISCUSSION

Volumetric analysis is a common diagnostic method in advanced neuroimaging. In human medicine, it is fully automated and contributes to the diagnosis of many neurological diseases.^19^, ^20^ The group of dogs consists of a large variety of breeds with different skull anatomies,^21^ which made automation of volumetric analysis challenging. This study attempted to automate the volumetric analysis of the central nervous system in dogs. Automated volumetry has a much higher sensitivity than manual volumetry, especially regarding the localization of small intracerebral anatomical structures.^22^

Although most dogs included in the study showed generalized seizures, epilepsy can have different phenotypes, which might often be atypical, such as behavioral disorders, focal twitching, or brief loss of consciousness.^23^, ^24^ Clinically, these episodes are often diagnosed as compulsive‐obsessive or other behavioral disorders, and their differentiation as well as confirmation of an epileptic background is made possible using EEG examination, which additionally allows us to localize the discharges. Temporal lobe epilepsy in humans often has a focal onset with secondary generalization.^25^, ^26^ However, early focal seizures in dogs often go unnoticed.^27^ In dogs with generalized epileptic seizures and epileptic discharges on the side of hippocampal atrophy, the focal onset of seizures cannot be ruled out. In humans, there are 2 types of temporal epilepsies: mesial TLE and lateral TLE. The former involves the mesolimbic part of the temporal lobe with epileptic discharges occurring within the hippocampus. The pathogenesis of temporal epilepsy is associated with hippocampal sclerosis caused by neuronal loss and astrogliosis.^28^ The pathogenesis of temporal epilepsy has been linked to sclerosis of the hippocampus because of neuronal loss and astrogliosis.^28^ According to the available literature, an asymmetry rate of more than 6% is considered significant in temporal epilepsy. It is estimated by assessing the volume of the hippocampus. For this reason, our study also assumed a significant factor of more than 6% in the temporal lobe measurements, because of changes in hippocampal volume because of TLE.^17^, ^18^ In the present study, 39% (12/31) of dogs with suspected idiopathic epilepsy with EDs in the temporal region had AR >6%, which may indicate structural changes in the brain. In the present study, 39% (12/31) of the dogs with suspected idiopathic epilepsy with epileptic discharges on the temporal region had an AR >6%, which could indicate structural changes in the brain. Additionally, 7 of these dogs showed a correlation between the EEG discharge side and temporal lobe volume reduction on the same side. Furthermore, 6 out of these 7 dogs had apparent ventricular asymmetry as indicated by AR on MRI which correlated with a decreased temporal lobe volume and EDs in its range on the same side. This may indicate a probability of TLE because of a reduction in the volume of the hippocampus and thus, the entire temporal lobe, and a widening of the ventricular system secondary to the lesions. Whereas volumetry of the hippocampus and ventricular system would be required for confirmation. Notably, 1 of these 7 dogs showed decreased temporal lobe and lateral ventricle volumes on the discharge side. This may be related to the presence of discharges on the contralateral side that were not recorded in the standard 30‐minute EEG study protocol. In addition, the ventricular asymmetry in these cases may have been secondary to seizures, and no lesions were observed in the MRI examination. In this case, it would also be interesting to extend the diagnosis with a volumetric study of the other brain lobes, as the asymmetry may be because of differences in the volumes of the other structures within the cerebral cortex.^29^, ^30^

In both human and veterinary medicine, hippocampal sclerosis, which can be associated with the presence of EEG discharges in the range of both temporal leads, can occur bilaterally,^31^ and in 30% of these patients, epilepsy is drug‐resistant.^32^, ^33^ The treatment methods of choice for refractory epilepsy include neurosurgery or vagus nerve stimulation.^34^, ^35^ Volumetric analysis during the course of drug‐resistant epilepsy can facilitate the decision toward the aforementioned surgical treatment method. The method described here can be a helpful and rapid diagnostic tool for drug‐resistant temporal epilepsy. This allows better localization of the epileptogenic focus, which will facilitate the planning of surgical procedures, such as temporal lobectomy. Automated volumetric analysis and its correlation with EEG results have the potential for therapeutic management of dogs with drug‐resistant epilepsy.

Three out of the 5 dogs from the group in which the AR ratio did not correlate with the side of discharges showed decreased temporal lobe volumes with increased volumes of the lateral ventricles opposite to the side of discharges. This result can indicate the presence of an additional epileptogenic focus on the contralateral side that was not detected in the 30‐minute EEG protocol. The animals can exhibit bilateral TLE with larger lesions on the side on which the discharges were not recorded.

In this study, no statistically significant differences were observed among Groups A, B, and C. This can be related to the lack of significant differences in temporal lobe volumes despite epileptic discharges. Because of the reproducibility of the results obtained, we believe that the automated method of measuring temporal lobe volumes is a good tool for assessing anatomopathological changes within the brain. There were statistically significant differences in AR between Group 2 (larger right ventricles) and Group 3 (symmetrical ventricles). Despite the lack of statistically significant differences, the AR in Group 1 was larger than that in Group 3.

There were no statistically significant differences in the temporal lobe size between Groups 1, 2, and 3 despite the apparent differences in the ventricular size. This could be related to the fact that no substantial changes in the temporal lobe volumes were observed despite the apparent asymmetry. It should be noted that most of the animals were diagnosed at the onset of epileptic seizures. The results obtained could also be because epilepsy is associated with sclerotization of the hippocampus in some animals and necrosis of the hippocampus in others. In addition, it could be related to the small size of the control group and high individual variability within the *Canis familiaris* species.

The greatest strength of the method used in this study is full automation, which eliminates the human error of inaccurately marking regions of interest.^36^ Owing to the small size of the investigated structures and the difficulty in localizing these structures in standard structural MRI, it was not possible to assess the temporal lobe accurately using a manual method. The described methodology can be used to assess temporal lobe volumes and helps better understand the pathogenesis of idiopathic epilepsy in both animals and humans.^37^, ^38^, ^39^

Approximately 30% of focal epileptogenic lesions are missed on standard MRI in human medicine, and advanced neuroimaging modalities are required in these cases.^13^ Extrapolation of these data to the veterinary field could lead to an alarming conclusion that dogs with normal blood tests and no identifiable structural lesions on MRI can be diagnosed with idiopathic epilepsy, when in fact they could have a temporal lobe lesions underlying the seizures. This changes the approach to idiopathic epilepsy in dogs, especially drug‐resistant epilepsy. There is a certain percentage of dogs with refractory epilepsy, and some of these dogs have structural changes, such as hippocampal sclerosis or cortical dysgenesis, within the brain. In such cases, volumetric analysis of the brain lobes and other specific structures, such as the hippocampus and amygdala, can be performed. This has potential for the development of surgical treatments in animals.

## CONFLICT OF INTEREST DECLARATION

Authors declare no conflict of interest.

## OFF‐LABEL ANTIMICROBIAL DECLARATION

Authors declare no off‐label use of antimicrobials.

## INSTITUTIONAL ANIMAL CARE AND USE COMMITTEE (IACUC) OR OTHER APPROVAL DECLARATION

In accordance with the Experiments on Animals Act from January 15th 2015. Ethical review and approval were not required for this animal study because this is a retrospective study from previously obtained clinical data. All methods were performed in accordance with relevant guidelines and regulations.

## HUMAN ETHICS APPROVAL DECLARATION

Authors declare human ethics approval was not needed for this study.

## Supporting information


**Data S1.** Supporting Information.
